# An Enhanced Rate-Based Emission Trading Program for NO_x_: The Dutch Model

**DOI:** 10.1100/tsw.2001.353

**Published:** 2001-12-01

**Authors:** Anne M. Sholtz, Bill Van Amburg, Verne K. Wochnick

**Affiliations:** Automated Credit Exchange, Pasadena, CA 91105, USA

**Keywords:** emission, emission trading, compliance, compliance planning, nitrogen oxide, NOX, nitrogen, eutrophication, cap and trade, rate-based, VROM, Ministry of Housing, Spatial Planning and the Environment, performance standard, performance standard rate, PSR, Netherlands, ACE, Automated Credit Exchange, pollution

## Abstract

Since 1997 government and industry in The Netherlands have been engaged in intensive policy discussions on how to design an emission trading program that would satisfy the Government’s policy objectives within the national and international regulatory framework and accommodate industry’s need for a flexible and cost-effective approach. Early on in the discussion the most promising solution was a rate-based approach, which dynamically allocated saleable emission credits based on a performance standard rate and actual energy used by facilities. All industrial facilities above a threshold of 20 MWth would be judged on their ability to meet this performance rate. Those “cleaner” than the standard can sell excess credits to others with an allocation that is less than their actual NO_X_ emission. With some changes in law, such a design could be made to fit well into the national and EU legislative framework while at the same time uniquely meeting industry’s requirement of flexibility toward economic growth and facility expansion. (An analysis of the legislative changes required will be given in a separate paper by Chris Dekkers.) However, the environmental outcome of such a system is not as certain as under an absolute emission cap. At the request of the Netherlands Ministry of Housing, Spatial Planning and the Environment (VROM), Automated Credit Exchange (ACE), in close cooperation with the working group of government and industry representatives introduced a number of features into the Dutch NO_X_ program allowing full exploitation of market mechanisms while allowing intermediate adjustments in the performance standard rates. The design is geared toward meeting environmental targets without jeopardizing the trading market the program intends to create. The paper discusses the genesis of the two-tier credit system ACE helped to design, explains the differences between primary (fixed) and secondary (variable) credits, and outlines how the Dutch system is expected to function once implemented in 2004. The paper also discusses the market trading simulation held in early 2001 to assess and test the trading program, and reviews also the current status of the market program development.

### INTRODUCTION

Despite a 70% reduction in $SO_X$ emissions through the 1980s and 1990s, the Netherlands continues to face a significant problem in atmospheric-based acidification. This problem remains driven by oxides of nitrogen ($NO_x$) and ammonia. $NO_x$ also contributes to eutrophication and is a precursor for ground-level ozone formation. Historic efforts to reduce such emissions have been limited, notably due to emissions from mobile sources and from the agriculture sector, but also from the industrial sources that did not meet their targets. Traditional regulatory approaches were no longer effective. This fact resulted in a joint undertaking by government and industry to seek new approaches to solving the $NO_X$ problem. Looking to achieve a more cost-effective approach, the Dutch Ministry of Housing, Spatial Planning, and the Environment (VROM) embarked on an effort to create a system of emission trading. However, from the beginning the Dutch were committed to developing a system that lacks the inherent limitations of existing cap-and-trade systems that have been employed in other countries, such as the U.S. One limitation is that hard cap-and-trade systems inhibit new facilities from entering such regional markets by forcing them to pay the penalty of purchasing needed offsets from existing industry. Another equally important limitation involves the concept of Best Available Technology (BAT) and the requirement by national and EU legislation to incorporate the "legal" incentive to achieve emission as low as reasonably possible. The cap-and-trade program does not incorporate that legal requirement, although its effect may be the same.

Therefore, the Netherlands decided to develop a trading instrument that would permit free entry into the economy while still addressing the need to reduce $NO_X$ emissions within the region. Such a system would be based on a standard, or uniform, rate of emissions to which all facilities, new or old, would be subject. However, while such a system can greatly increase flexibility by accommodating economic expansion, it also presents unique challenges, not the least of which can be a reduced certainty of environmental outcome.

VROM retained the Automated Credit Exchange™ (ACE) of California, which has expertise in designing and operating regulatory emission trading programs, to provide analyses, predesign studies, development, and simulation of a trading system based on a performance standard for the industrial sources in the Netherlands.

### BACKGROUND FOR PERFORMANCE STANDARD RATE SYSTEM

The Dutch government, in collaborative efforts with industry working groups, has since 1997 been engaged in developing a new, innovative, and industry-responsive mechanism that can provide $NO_X$ reductions in line with government short- and long-term objectives while maintaining flexibility and cost effectiveness for facility emission controls. The Netherlands discovered that it was reaching the limits of progress that could be made with more traditional "command and control" regulatory schemes, or with industry agreements. Emission credit trading was perceived to be a market-based approach that could provide the next level of reductions required.

Emission trading programs offer certainty of environmental outcome while creating compliance and operational flexibility for market participants. Programs can also be designed to encourage the development of new and cleaner technology. In contrast, the use of taxes on sources as a strategy cannot guarantee a quantitative outcome and must be continually adjusted; however, command-and-control regulations can achieve emission limits, but at the cost of greatly reduced flexibility and increased administration.

Additionally, for facilities facing excessive control costs, trading programs can provide an alternative "control" option–purchasing credits from facilities that may have much lower costs of control while maintaining the required overall emissions reductions. As attractive as that general theory of emission trading appears, the Dutch faced specific constraints and needs in any emission trading program they were to develop. Such a program would need:

- To meet the needs of the Netherlands government to control acidification, ozone, and eutrophication resulting from $NO_X$ emissions;
- To meet the needs of industry to have a cost-effective mechanism for meeting environmental requirements;
- To meet the EU and other international environmental requirements, including the UN Economic Commission for Europe (UN-ECE) Convention on Long-Range Transboundary Pollution; and
- To accommodate the European Union's requirements for "freedom of establishment" — meaning the freedom for any European company to set up business in any EU country with the minimal restrictions.

The rate-based system, as developed by the Netherlands, was intended to be compatible with existing legislation, though it is now clear some modifications of environmental law will be required to implement the system (a complete discussion of this is found in the paper by Chris Dekkers, www.thescientificworld.com). Importantly, in the Dutch and European perspective, the rate-based system accommodates new entrants without any penalty or perception of preference and more easily accommodates existing facility expansion and economic growth. The reason for this is how it allocates credits, or emission rights, to participants.

In the "cap-and-trade" model of emission trading, regulators provide fixed emission "allocations" in the form of credits to all participants at the program's inception. Later arrivals, such as new or transplanted firms, must acquire their credit allocation, or emission rights, from existing facilities. This sets a barrier to new entry and a preference for existing firms. This is incompatible with EU directives. The rate-based model, by contrast, sets its allocation dynamically based on the performance of the facility. All facilities are subject to the standard and are measured against it. Any new facility can establish operations and receive an emission credit allocation, but it is subject to the emissions performance standard.

At the same time, because of the flexibility of its design to accommodate economic growth, a rate-based system also brings with it some additional uncertainty compared with a cap-and-trade structure. Because the Netherlands program establishes a rate of emissions per unit of energy, future rates established under the program will be highly dependent on accurate forecasts of future energy capacity and economic growth. Should those forecasts be incorrect, rate adjustments may be required to maintain environmental goals. A mechanism is therefore needed to accommodate any rate adjustments that may be necessary to meet this target commitment.

The compensating mechanism that has been developed is a two-tier credit system, utilizing a primary and secondary allocation[1]. It will be outlined in the following sections. This market-based tool accommodates rate adjustments and allows market participants to separately price the risk of any adjustment.

### SYSTEM DESIGN AND APPROACH

#### Overview

In broad terms, the Netherlands program concept is built upon a rate-based structure, designed around a declining Performance Standard Rate (PSR). This rate is the "standard of performance" for each individual facility's emissions, and it is derived from two main variables: the overall target emissions level for the trading group, divided by the projected energy produced by the group. The PSR will follow a yearly decline toward the target of 2010, which represents roughly a 50% reduction compared with 1995 emissions.

The PSR is expressed as a series of performance "ratios" of emissions per energy unit. The scale chosen to express this is grams of $NO_X$ per gigajoule of energy (g/GJ)(Fig. 1). Based on existing agreements for $NO_X$ emission targets for the year 2010, and on projections of energy produced by the industrial sector, the Dutch are aiming toward a PSR of 50 g/GJ in 2010.

In practice, there will be several "PSRs" implemented to accommodate specific processes in the Netherlands. The main series of PSRs, discussed above, will apply to combustion emissions, which account for the significant majority of $NO_X$ emissions— more than 80%—from those facilities that will be affected by the program. There will also be PSRs assigned to specific processes, including the production of steel, zinc, aluminum, nitric acid, soot, and magnesium dioxide. These processes will be subject to an overall 40% reduction from 1995 levels by 2010.

For regulatory manageability, the program will only apply to facilities that produce more than a threshold level (20 MWth/year) of energy in their operation. Such an approach is effective, however, because the roughly 200 facilities subject to the program together emit more than 85% of the industrial $NO_X$ in the Netherlands. Facilities using fossil fuel to produce energy are included in the system. Facilities that only use energy produced by others (such as electricity usage) are not in the program.

The following are key highlights of the program, beginning with its basic premises:

- Rate Based: the system will be structured around an allowable rate of emissions, known as a PSR, with the actual PSRs determined by the government.
- Milestone Year(s): to measure the effectiveness of the overall program, one milestone year prior to 2010 (proposed for 2006) will be established to allow the government to assess whether the emission target for 2010 will be met. If the program proceeds beyond 2010, then 2010 itself may also be a milestone year.
- Two Credits: to accommodate the possibilities of future changes in the PSR, the trading system will include two tiers of credits: primary and secondary. Primary credits will be those whose basis for allocation—the primary portion of the PSR¾cannot be changed or devalued. They would constitute,

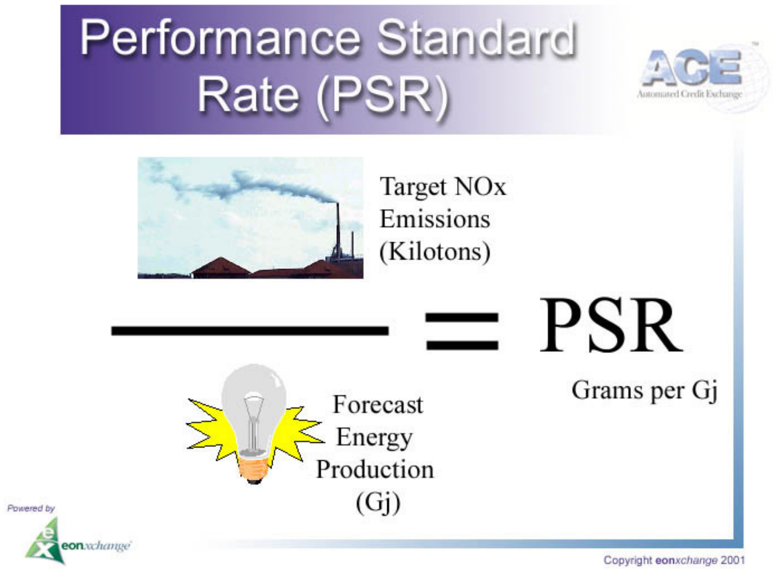

**FIGURE 1.** Performance standard rate.

in most instances, the majority of the total credit allocation. Secondary credits will be those whose basis of allocation may change — or be devalued — if the PSR changes and therefore carry a risk of reduction in emission compliance value. The marketplace then assesses and prices this risk of devaluation.

- PSR Adjustment: the responsibility for setting the PSR basis for these credits, and for any adjustments to the PSR, rests with the government. Once a PSR is adjusted, following a milestone year evaluation, secondary credits in years following the milestone will be devalued by an amount that compensates for any unreached emission target.
- Two Cycles: to ensure that trading is not all segregated to one time of the year—thus easing the risk of price "spikes"––there will be two "cycles" of trading, similar to the RECLAIM (Regional Clean Air Incentive Market) system in Southern California. Roughly half the program's emissions will be represented in a calendar year trading cycle (January 1–December 30), and half in a fiscal year (July 1–June 30) trading cycle. Companies may use credits from either "cycle" for reconciliation purposes, as long as those credits "overlap" in time with the emissions.

Under this system, then, each facility will determine its emissions allocation yearly based on its actual performance and will then compare this against its actual emissions during that same period to determine compliance, i.e., whether it is meeting the PSR (Fig. 2).

To determine compliance, a company will take its energy production in gigajoules and multiply it by that year's PSR. The result is its allocation: an amount of emissions, in grams (or more likely converted to kilograms) $NO_X$. In simple terms, a facility whose actual emissions exceed its allocation will effectively have a negative balance and will need to purchase additional credits. Conversely, in a year when a facility's actual emissions are less than the PSR-determined allocation, the facility will be in a position to sell excess credits (Fig. 3). Future year sales and purchases of credits will be based on a facility's projected energy production.

### The Secondary Credit

It is possible to exceed the target total emissions even though each company is in full compliance with the PSR. If such an unanticipated growth in energy production is found to have occurred following a milestone year, a downward adjustment of the PSR is necessary to achieve the national emission ceiling defined by the Dutch government (Fig. 4). The possibility of such adjustments creates uncertainty within the system and can stifle economic planning if dealt with improperly—hence the need for some guarantees via two credit types, only one of which can be devalued.

Providing this separate secondary credit allows facilities to price the perceived risk of any future changes and use the market to allocate that risk most efficiently across facilities (Fig. 5).

Potential PSR adjustments would translate into reductions in secondary credits via a secondary credit discount factor, which would be established in advance (Fig. 6).

Facilities can sell, buy, and/or swap primary or secondary credits. Secondary credits would not face adjustments until milestone years and then only from that milestone year forward.

### Secondary Credit Application

A framework for the PSR has been broadly agreed to after much discussion between industry and government. Its endpoint is the industrial sector's portion of the 2010 $NO_X$ emissions requirement in Dutch law and exceeding EU standards, combined with

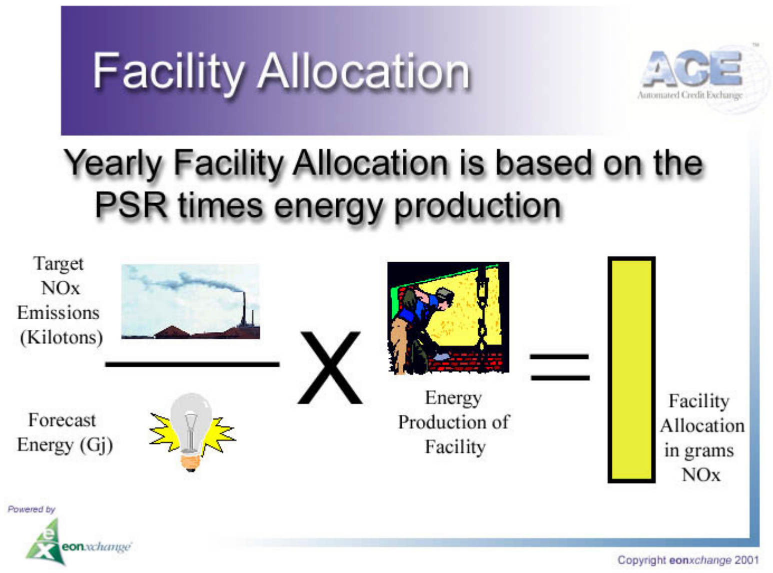

**FIGURE 2.** Facility allocation.

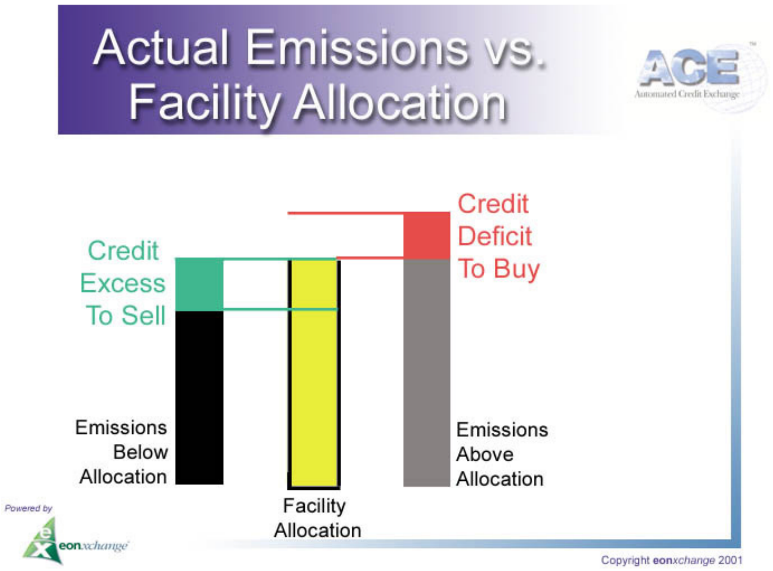

**FIGURE 3.** Actual emissions vs. facility allocation.

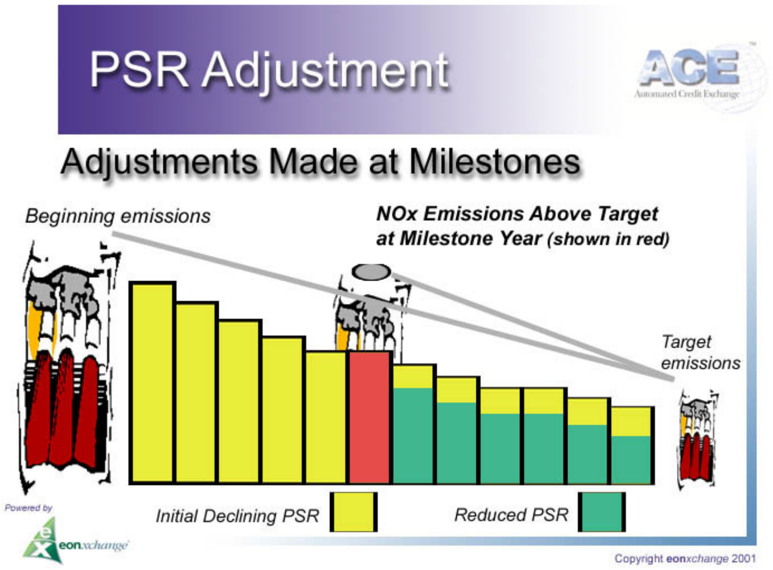

**FIGURE 4**. PSR adjustment.

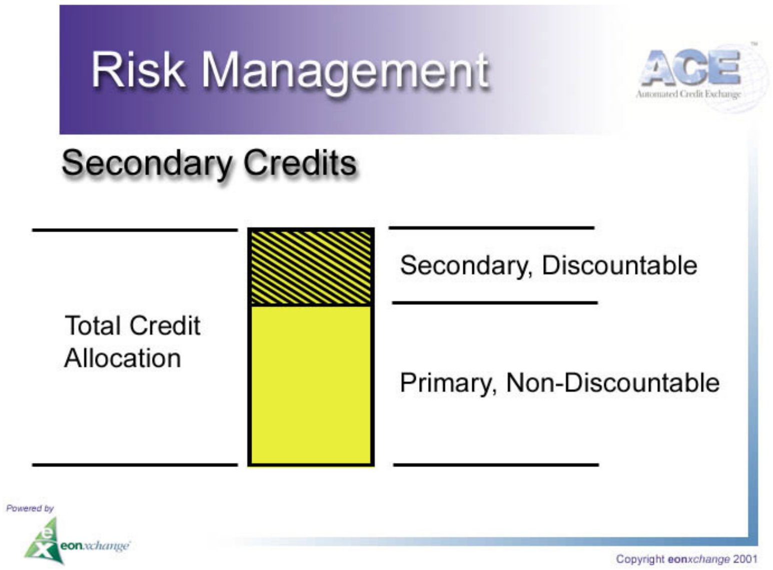

**FIGURE 5.** Risk management.

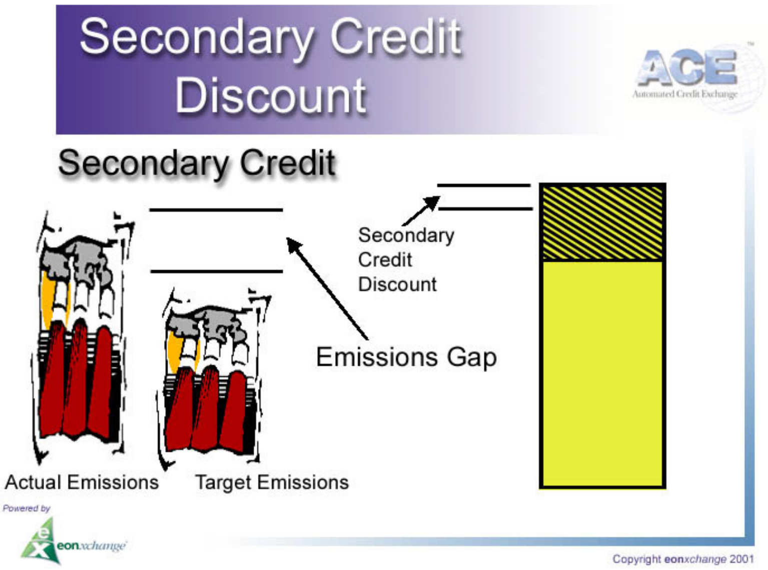

**FIGURE 6.** Secondary credit discount.

the assumed energy production of the industrial sector by that period. This equates to an overall PSR of 50 g/GJ in 2010. The starting point is based on 1995 emissions and energy production for this same segment. In 1995, the average emission rate equated to 105 g/GJ.

Determining the "slope" of the declining PSR between these points is still under development. Also under discussion is what portions of the total PSR allocation will be considered primary and secondary, although the ultimate proportion might be in the 80/20 or 75/25 range of primary to secondary. An example of an approach, as used in a recent simulation, is found in Table 1. In this example, by 2010, 40 g/GJ is the primary component of the combustion-source PSR, with 10 g/GJ being the secondary component. If in 2006 a PSR adjustment were to be required and therefore secondary credits were devalued, only the 10 g/GJ portion would be subject to change. In essence, this means that the greatest possible reduction that could be implemented would be to the 40 g/GJ level by 2010 (if all secondary credits were devalued).

Secondary credits can be purchased at any time in future years as part of the trading system. They only have compliance value in the specific years for which they are issued. In practical terms, secondary credits provide an additional level of responsibility as well as compliance flexibility for users. A portion of the total allocation, 20% by 2010, is secondary and in theory discountable depending on the overall success of the program. Participants will want to closely track the overall energy use and total yearly emissions of the program group—as reported by the regulatory authorities—and then compare this with their own risk profile, control of costs, and flexibility of costs. In general

**TABLE 1**
**Primary and Secondary PSRs for Maastricht Simulation**

| Year | PSR | Primary Part | Secondary Part |
|---|---|---|---|
| 2002 | | | |
| 2003 | 95 | 95 | — |
| 2004 | 88 | 88 | — |
| 2005 | 82 | 82 | — |
| 2006 | 75 | 75 | — |
| 2007 | 68 | 58 | 10 |
| 2008 | 62 | 52 | 10 |
| 2009 | 56 | 56 | 10 |
| 2010 | 50 | 40 | 10 |
| 2011 | 50 | 35 | 15 |
| 2012 | 50 | 30 | 20 |

*Note:* All figures shown are in grams of NOx emission per gigajoule of energy production.

terms, a company with low and relatively flexible emission control options (meaning those that can be implemented quickly, or easily expanded) would face less risk from secondary credit devaluation than a company with high cost and/or inflexible control options (such as those requiring long lead times or providing only fixed reductions). Such companies would probably value secondary credits differently, thus allowing them to use this additional market mechanism as a way to better manage that risk[2].

The program leaves the decision of how best to trade secondary credits up to the individual market participant. For instance, a facility could sell all of its expected secondary credit allocation in future years and purchase only primary credits, thereby eliminating the devaluation consequences of a PSR adjustment on its allocation—presumably at a cost. Conversely, a facility could hold its secondary credits and trade primary credits, thereby realizing greater profits but bearing greater risk from a PSR adjustment. These simple examples show the additional market-based risk management available to market participants with the two-credit approach.

### Two Trading Cycles

Tradable credits will expire annually and will be issued as one of two cycles, distributed in relatively equal proportions across the cycles. Cycle 1 credits may be used from January 1 through December 31 of a given year. Cycle 2 credits may be used for the period of July 1 through June 30 of the following year (Fig. 7). The regulatory agency could additionally identify cycle 1 and cycle 2 facilities for the purposes of spreading administrative burden across the year. Regardless of cycle designation, facilities will be able to use either cycle for compliance. Facilities would be randomly assigned to cycles, with roughly half the emissions assigned to each.

The two-cycle approach allows for free substitution of credits between the cycles, thereby stabilizing the price of credits at year-end. A 1993 study by the California Institute of Technology (CALTECH), in conjunction with the Pacific Stock Exchange, conducted for the emission regulatory authority in South- e r n California (the South Coast Air Quality Management District—SCAQMD), showed that if all credits were to expire simultaneously, the price of expiring credits would swing wildly, depending upon facility emissions revealed only at the end of the year[3]. In order to counteract the problems inherent in this uncertainty, facilities would likely try to hold an excess of "insurance" credits until the end of the year, creating a barrier to economic growth in the region.

The two-cycle approach, therefore, is used to remove this problem. For example, at the end of its compliance year in December 2005, a cycle 1 facility, with a shortage of credits, would have the flexibility to purchase either expiring 2005 cycle 1 credits or 2005 cycle 2 credits. Conversely, at the end of its compliance year in June 2006, a cycle 2 facility, with a shortage of credits, would have the flexibility to purchase either expiring 2005 cycle 2 credits or 2006 cycle 1 credits.

Given this inherent flexibility of compliance, companies should therefore face penalties for noncompliance at the end of their cycle. The Dutch government has not yet established a penalty structure. By way of example, in the Maastricht simulation exercise, companies determined noncompliant were assessed a penalty that was deducted from future year credits: 1.2 times their "shortfall" in credits reduced from their next year's allocation. A second year of noncompliance resulted in a penalty of 1.5 times the shortfall being deducted from the following year.

## TESTING AND SIMULATION

### Organization

Once the framework for the emission trading program was established, it was deemed important to provide a "hands-on" demonstration of how it operates and how companies could use emission trading to make compliance decisions (Fig. 8). This

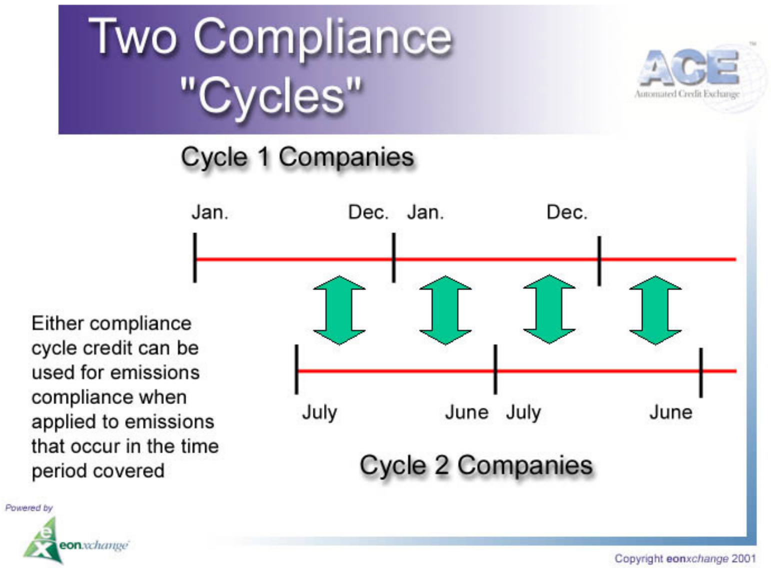

**FIGURE 7.** Two compliance cycles.

was of increased importance in the Netherlands and Europe because emission trading itself was a new process.

Over 2 days from February 7–8, 2001, more than 125 participants from 50 different companies and agencies participated in an emission trading simulation exercise. The simulation was staged at Maastricht, a city in the southern part of the Netherlands, and included most of the larger firms who will be subject to the $NO_X$ program together with representatives of key government agencies, private institutions, and industrial organizations who will play a role.

Each participating organization was provided with a "profile" for the simulation. For the 34 actual companies who participated, that profile was based on their own projections of emissions, energy production, and manufacturing for a 10-year period. In addition, each of these companies identified its various control strategies, including costs and reduction value. These were provided to them in their profiles as "options" they could choose to implement during the simulation. The 16 additional participants were assigned "virtual" profiles, based on realistic operating parameters and options of companies operating in the Netherlands.

Part of the challenge of the simulation development process was the realization that many companies do not have the data to make these projections; the simulation served as a useful planning exercise, but also showed an information gap on the part of companies that would have to be addressed. Effective emission trading requires effective planning.

Six years of trading markets and compliance periods were simulated during the exercise, allowing participants to weigh credit purchase or sale against their costs of control. They were also able to see how the process of "reconciliation" coming at the end of a compliance cycle works, and received reports on the overall emissions of the group compared with yearly goals. Compliance assessment, penalty, monitoring, and credit registry functions were all simulated, using the emission trading program design, by the Automated Credit Exchange (ACE), with KPMG Environmental. ACE also modified its electronic emission exchange platform to accommodate the Dutch program design for real-time trading.

The goal of this exercise was not to provide absolute market results and projections. Rather, it was intended to be a threefold learning experience:

- Provide a hands-on experience with emission trading
- Provide early preparation for the impending $NO_X$ program
- Answer questions about the program

### RESULTS

On the core issues listed above the simulation served an extremely useful and timely purpose. It is often difficult to visualize how policy objectives and program designs will "play out" without an opportunity to set the components in motion and see where questions are raised and problems develop. In the case of the Netherlands system, it was clear that most participating companies quickly began to use the emission marketplace in an effective and economically rationale way.

The market results showed, for instance, that overall the market price of credits reflected the steady implementation of the most cost-effective emission reduction strategies. These were not just the most effective strategies for individual companies, but those most effective (in volume of $NO_X$ reduced per Euro spent)(Fig. 9) for the entire industry group. Companies with the lowest cost strategies implemented their programs and sold credits, generally paying for the option with their credit sales, while providing lower cost strategies to others through these credits.

It is quite interesting to note that analysis of the data generally showed a very efficient and rationale market operation and

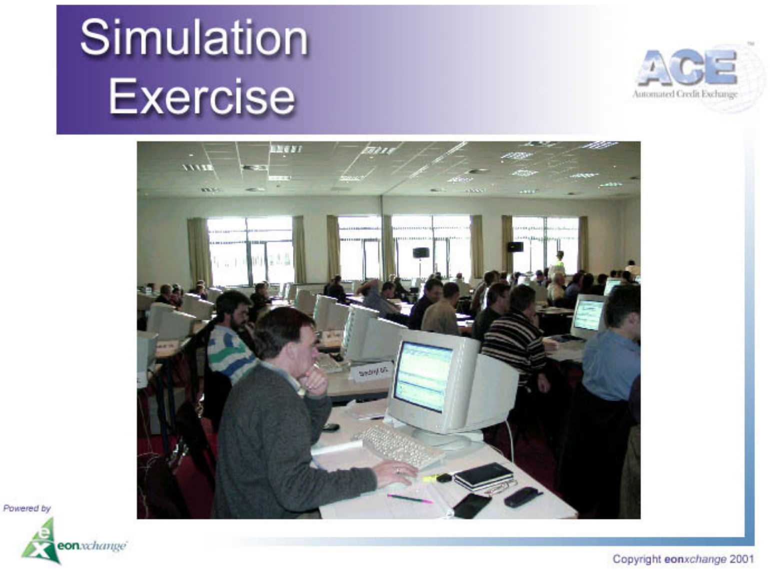

**FIGURE 8.** Simulation exercise.

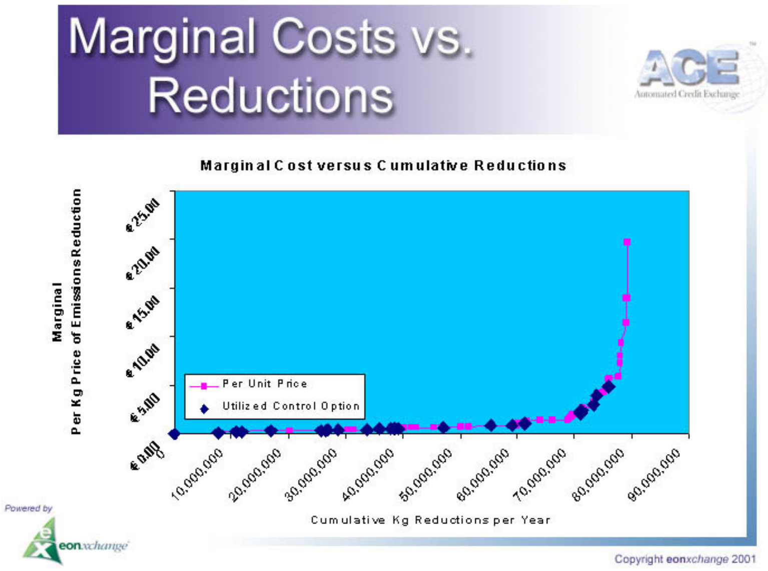

**FIGURE 9.** Marginal costs vs. reductions.

market responses. For instance, the average discount applied by companies to secondary credits was 32%; they paid nearly a third less for secondary credits than for primary. The implication is that companies felt a devaluation of secondary credits by 32% was likely. Further analysis uncovered that if such devaluation had occurred, there would have been a significant volume of additional emissions to reduce. Most notably, the marginal cost to implement that next incremental level of emission controls matched almost exactly the weighted average cost of credits in the market. This correlation shows a very efficient market process and intelligent economic choices being made by "traders."[4]

It also became clear that information reporting on the progress of the program is highly important. While the simulation did not forecast the expected absolute market results, it did provide a good behavioral learning model. In this first exercise, participants were expecting a greater-than-realistic devaluation in secondary credits—meaning missing the emission targets—than was actually the case. In point of fact, no devaluation was required because the emission target was easily met. However, participants either did not believe or were unsure how to interpret the program status reports delivered during the simulation. Clear trend information will be vital to this risk assessment, and, of course, effective emissions monitoring is critical for compliance and information purposes.

If there was one other key lesson of the simulation exercise, it was that the general group that seemed to be least effective at emission trading comprised participants from regulatory and other agencies. This is not meant as a snide observation, but one more illustrative of the change in thinking required for an effective new regulatory approach. Many of the government participants instantly enacted their control options, many much more expensive than equivalent credits would have been, at the beginning of or early into the simulation—before these options were necessary either for compliance or because of any market price signals. Enacting a technical solution despite the cost counters the benefits of a market mechanism and shows the change in mindset and understanding necessary to switch from the proscriptive regulatory approach to a greater trust of, and reliance in, a dynamic regulatory marketplace.

### CONCLUSIONS

The proposed Dutch emission trading program has so far proved its basic design concept and demonstrated that a rate-based approach can function as an efficient method of cost-effective emissions reduction while accommodating economic growth. One of the key concerns of a rate-based model—that it could provide certified environment results—has been solved through the use of a market-based mechanism that can buffer the market from the full shock of possible rate adjustments.

As such, the Dutch model is becoming a model for others of a regulatory instrument capable of meeting many of the emerging challenges of issues such as global warming and greenhouse gas (GHG) trading. For example, a rate-based approach eliminates concerns about receiving credit for "early action" in reducing emissions. Companies that have already made significant reductions would simply perform better against the performance standards than companies that have taken no action. It is also geared toward greater efficiency and cleaner fuels and technologies with its focus on reducing emissions per unit of energy or production.

While not currently planned for the program, these authors opine that "alternative" energy (such as wind, hydro, solar, and geothermal) producers could also be accommodated in such a program. Since these power production techniques consume no fossil fuel, one might measure only their energy output to determine their allocation. Because of their substantial emissions benefits, their potential source of low control-cost credits, and the market-based incentive that credits provide low or zero $NO_X$ alternatives, it could be an attractive device to accelerate green energy production.

The rate-based system's variable, yearly allocations add complexity to the trading system and require facilities to better understand and plan for future actions. The availability of future credits is impacted by both a facility's expected performance and by potential changes in the value of secondary credits.

However, the rate-based allocation process removes many barriers to entry for new or expanding facilities and can broaden the trading group and market. Under the rate-based system, there would be no program barriers for new facilities to open. New, clean firms will not be required to purchase a starting allocation. Indeed, if a new facility utilized state-of-the-art clean technology, it could actually generate excess credits from the moment of its operation.

The Netherlands program has weathered many tests to date, not the least of which has been maintaining the unique cooperative ties between Dutch industry and government, creating an open atmosphere of mutual trust, and willingness to compromise and experiment. Many challenges loom ahead, however, including the required changes in Dutch and EU legislation to enable the program and setting the subsequent annual declines of the PSR. As the program matures and begins to face the controversial and detail-based issues of agreeing to monitoring protocols, reporting standards, compliance penalties and the like, that collaborative commitment faces its toughest challenges yet.

| | | |
|---|---|---|
| Received: | July | 27, 2001 |
| Revised: | October | 10, 2001 |
| Accepted: | November | 1, 2001 |
| Published: | December | 1, 2001 |

### BIOSKETCH

**Bill Van Amburg** is Senior Vice President of the Automated Credit Exchange (ACE), where he manages environmental applications. He managed the Netherlands $NO_x$ Emission Trading Development program performed for the Dutch government, which provided the framework for and a simulation of a rate-based emission trading system. He also has expertise in commercializing new technologies. Bill previously was vice president of America's leading advanced transportation technologies consortium (CALSTART), is an Emmy-award winning journalist, and is a graduate of the UCLA Anderson School's Executive Management Program. The author of several papers, he has a brand management certificate from Stanford and a Bachelors Degree in Anthropology from U.C. Berkeley.
